# Diabetic Foot Risk Classification at the Time of Type 2 Diabetes Diagnosis and Subsequent Risk of Mortality: A Population-Based Cohort Study

**DOI:** 10.3389/fendo.2022.888924

**Published:** 2022-07-11

**Authors:** Zhaonan Wang, Jonathan Hazlehurst, Anuradhaa Subramanian, Abd A. Tahrani, Wasim Hanif, Neil Thomas, Pushpa Singh, Jingya Wang, Christopher Sainsbury, Krishnarajah Nirantharakumar, Francesca L. Crowe

**Affiliations:** ^1^Institute of Applied Health Research, University of Birmingham, Birmingham, United Kingdom; ^2^Department of Diabetes and Endocrinology, University Hospital Birmingham Foundation Trust, Birmingham, United Kingdom; ^3^Centre for Endocrinology, Diabetes and Metabolism, Birmingham Health Partners, Birmingham, United Kingdom; ^4^Institute of Metabolism and Systems Research, University of Birmingham, Birmingham, United Kingdom; ^5^Department of Diabetes, Gartnavel General Hospital, National Health Service Greater Glasgow and Clyde, Glasgow, United Kingdom; ^6^Health Data Research UK, London, United Kingdom

**Keywords:** type 2 diabetes, diabetic foot risk, diabetic foot disease, mortality, foot risk examination

## Abstract

**Aim:**

We aimed to compare the mortality of individuals at low, moderate, and high risk of diabetic foot disease (DFD) in the context of newly diagnosed type 2 diabetes, before developing active diabetic foot problem.

**Methods:**

This was a population-based cohort study of adults with newly diagnosed type 2 diabetes utilizing IQVIA Medical Research Data. The outcome was all-cause mortality among individuals with low, moderate, and high risk of DFD, and also in those with no record of foot assessment and those who declined foot examination.

**Results:**

Of 225,787 individuals with newly diagnosed type 2 diabetes, 34,061 (15.1%) died during the study period from January 1, 2000 to December 31, 2019. Moderate risk and high risk of DFD were associated with increased mortality risk compared to low risk of DFD (adjusted hazard ratio [aHR] 1.50, 95% CI 1.42, 1.58; aHR 2.01, 95% CI 1.84, 2.20, respectively). Individuals who declined foot examination or who had no record also had increased mortality risk of 75% and 25% vs. those at low risk of DFD, respectively (aHR 1.75, 95% CI 1.51, 2.04; aHR 1.25, 95% CI 1.20, 1.30).

**Conclusion:**

Individuals with new-onset type 2 diabetes who had moderate to high risk of DFD were more likely to die compared to those at low risk of DFD. The associations between declined foot examination and absence of foot examinations, and increased risk of mortality further highlight the importance of assessing foot risk as it identifies not only patients at risk of diabetic foot ulceration but also mortality.

## Introduction

Diabetic foot disease (DFD) has been recognized as a significant clinical condition that causes hospitalization and morbidity in people with diabetes (1). Approximately 34% of patients with diabetes are likely to be affected by foot ulcers, and 20% of those require an amputation (2, 3). DFD also significantly worsens the quality of life in people with diabetes (4, 5). Notably, diabetic foot ulceration (DFU) and amputation were associated with increased mortality among people with diabetes (6–8). Approximately 50% of those developing a DFU and up to 70% of individuals with amputation die within 5 years in the UK (6). Although it aggravates the health burden and increases mortality in people with type 2 diabetes, DFD is preventable by early detection of foot risk and by implementing appropriate preventative care before the development of active foot disorders (9–11). For this reason, National Institute for Health and Care Excellence (NICE) guidelines recommend that all adults with newly diagnosed diabetes should have a foot examination (6). There are, however, still a large number of people with diabetes who do not have a foot examination either in primary or in secondary care (12, 13).

Diabetic foot risk including neuropathy, deformity, peripheral arterial disease, and history of ulcer or amputation has been highlighted to increase the risk of diabetic ulcer and amputation (9, 14, 15). However, evidence for an association of at-risk foot in the early course of type 2 diabetes, before the development of active foot disorder, with mortality is lacking. Evidence linking peripheral neuropathy at the time of diagnosis of type 2 diabetes with cardiovascular disease (16) or mortality (16, 17) is limited to modest-sized studies. In the UKPDS outcomes model, PVD, amputation, and ulcer are predictors of mortality (18) while available data of peripheral neuropathy do not form part of the model. It is worth considering the mortality of individuals with at-risk foot as it can identify individuals who are at greater risk of mortality at the time of diagnosis of type 2 diabetes, hence enabling implementation of preventative interventions to reduce the mortality in the long run. There is also a paucity of data describing the risk of mortality among type 2 diabetes patients who do not have a foot examination. We hypothesized that individuals with newly diagnosed type 2 diabetes who were at increased risk of DFD would be associated with higher risk of mortality. Highlighting at-risk foot as a significant indicator of death at the time of diagnosis of type 2 diabetes will ensure early intervention rather than at a later stage. Therefore, we aimed to compare the mortality of individuals at low, moderate, and high risk of DFD, and also those with no record and who declined foot examination in the context of newly diagnosed type 2 diabetes.

## Methods

### Study Design and Data Source

We conducted a population-based cohort study of individuals with newly diagnosed type 2 diabetes between January 1, 2000, and December 31, 2019 in the IQVIA Medical Research Data (IMRD). IQVIA, incorporating data from The Health Improvement Network (THIN), is a longitudinal, clinical primary care database of over 18 million patient records in the UK (19). Read codes describing concepts related to health in GP records are used to record diagnoses in the IMRD database (20). Collection for data in IMRD was approved by the NHS South East Multi-centre Research Ethics Committee (MREC) in 2003. We obtained approval to conduct this analysis from the Scientific Review Committee (reference number: 21SRC030).

The Data Extraction for Epidemiological Research (DExtER) tool, an extract transform load-based software framework, was used to extract this dataset (21). This platform enables users to extract high-quality and individual-patient-level data from primary care databases (21). The outcome measures (e.g., prevalence) calculated in IMRD datasets extracted from DExtER have produced comparable results to those from Clinical Practice Research Datalink (CPRD) and other national datasets (21).

### Study Population

Adults ≥18 years with a record of type 2 diabetes diagnosis and registered with an eligible practice for at least 1 year before study entry were eligible for the study. Type 2 diabetes diagnosis was ascertained by the presence of any type 2 diabetes clinical (Read) code in the individual’s medical record. Adults with a recording of type 1 diabetes were excluded.

### Exposure and Outcome Measures

The main exposure was the risk of DFD based on Read codes that have previously been used in a microvascular complications study (22). Based on NICE guidelines, risk of DFD was categorized into three groups—low, moderate, and high (6). We considered individuals with no evidence of diabetic peripheral neuropathy (DPN), no peripheral vascular disease (PVD), and no presence of foot deformity, impairment, or previous ulcer to be at low risk (6, 22, 23). Individuals presenting with deformity, neuropathy, or non-critical limb ischemia were considered to be at moderate risk (6, 22, 23). Previous ulceration, amputation, and more than 2 of 3 parameters of DPN, PVD, or deformity were defined as high risk (6, 22, 23). The outcome was all-cause mortality among those newly diagnosed with type 2 diabetes with low, moderate, and high risk of DFD.

### Follow-Up

The index date was defined as 15 months following the date of diagnosis of type 2 diabetes (24), which was chosen because of the requirement to measure foot risk soon after diagnosis of diabetes and reassess the risk annually as per NICE guidelines and Quality Outcomes Framework (QOF) in the UK (6, 25). The QOF indicator is defined as the percentage of patients with diabetes with a record of a foot examination and risk classification within the preceding 15 months (25). Follow-up started at the index date of 15 months post type 2 diabetes diagnosis and ended at exit date defined as the occurrence of one of the following events (whichever came earliest): (a) death, (b) individual left the practice, or (c) study end date (December 31, 2019). In an additional analysis, we took an index date of 30 months following diagnosis of type 2 diabetes, giving additional time for foot risk assessment to take place after a diagnosis of type 2 diabetes.

### Covariates

Baseline characteristics included age, sex, BMI (kg/m^2^), smoking status, ethnicity, social deprivation status, history of CVD, and HbA1c (mmol/mol). BMI was classified according to NICE BMI classification as follows: underweight (BMI of <18.5 kg/m^2^), normal weight (BMI of 18.5 to <25 kg/m^2^), overweight (BMI of 25 to <30 kg/m^2^), obesity class I (BMI of 30 to <35 kg/m^2^), obesity class II (BMI of 35 to <40 kg/m^2^), and obesity class III (BMI of ≥40 kg/m^2^) (25). Smoking status was categorized as smoker, non-smoker, and ex-smoker. Ethnicity was classified based on UK census ethnic groups (White; Black, African, Caribbean, or Black British; Asian or British Asian; mixed or multiple ethnic groups; and other ethnic groups). The Townsend deprivation index of social deprivation status was based on quintiles with 1 being the least deprived and 5 being the most deprived (26). CVD was defined as atrial fibrillation (AF), heart failure, ischemic heart disease (IHD), and stroke and transient ischemic attacks (TIA). HbA1c was categorized as ≤47.5 mmol/mol, 47.5–58.5 mmol/mol, 58.5–69.4 mmol/mol, and >69.4 mmol/mol (24). Drugs included lipid drugs, metformin, insulin, and other diabetes drugs (glitazones, glinides, acarbose, glucagon-like peptide 1 [GLP-1] receptor agonists, dipeptidyl peptidase-4 [DPP-4] inhibitors, sodium-glucose co-transporter-2 [SGLT-2] inhibitors, and sulfonylureas). Missing data for BMI, Townsend deprivation index, smoking status, ethnicity, and HbA1c were assigned to a separate category and included in the analyses.

### Statistical Analysis

In the analysis, means (± SD) were used to summarize continuous variables, and percentages were used to summarize categorical variables. Crude and adjusted HR and 95% CIs were calculated for the occurrence of death in DFD risk groups using a Cox proportional model. The log–log plots were used to check proportional hazards assumption with almost parallel curves indicating that the assumption was not violated. Baseline characteristics including age, sex, Townsend score, ethnicity, smoking status, BMI, CVD, HbA1c, and drug use were included as covariates in the regression model. Kaplan–Meier survival curves were generated for different DFD risk groups, and the log-rank test was performed to test the equality of the survivor function between groups.

Sensitivity analysis I involves setting the index date 30 months after diagnosis with type 2 diabetes, and was performed using the same statistical methods as in the main analysis; sensitivity analysis II concerns the exclusion of individuals who had incomplete data on BMI, smoking status, and Townsend score.

We considered 2-tailed *p*-value <0.05 to be statistically significant. All statistical analyses were conducted using Stata version 16 software.

## Results

### Study Population Characteristics

**Figure 1:**
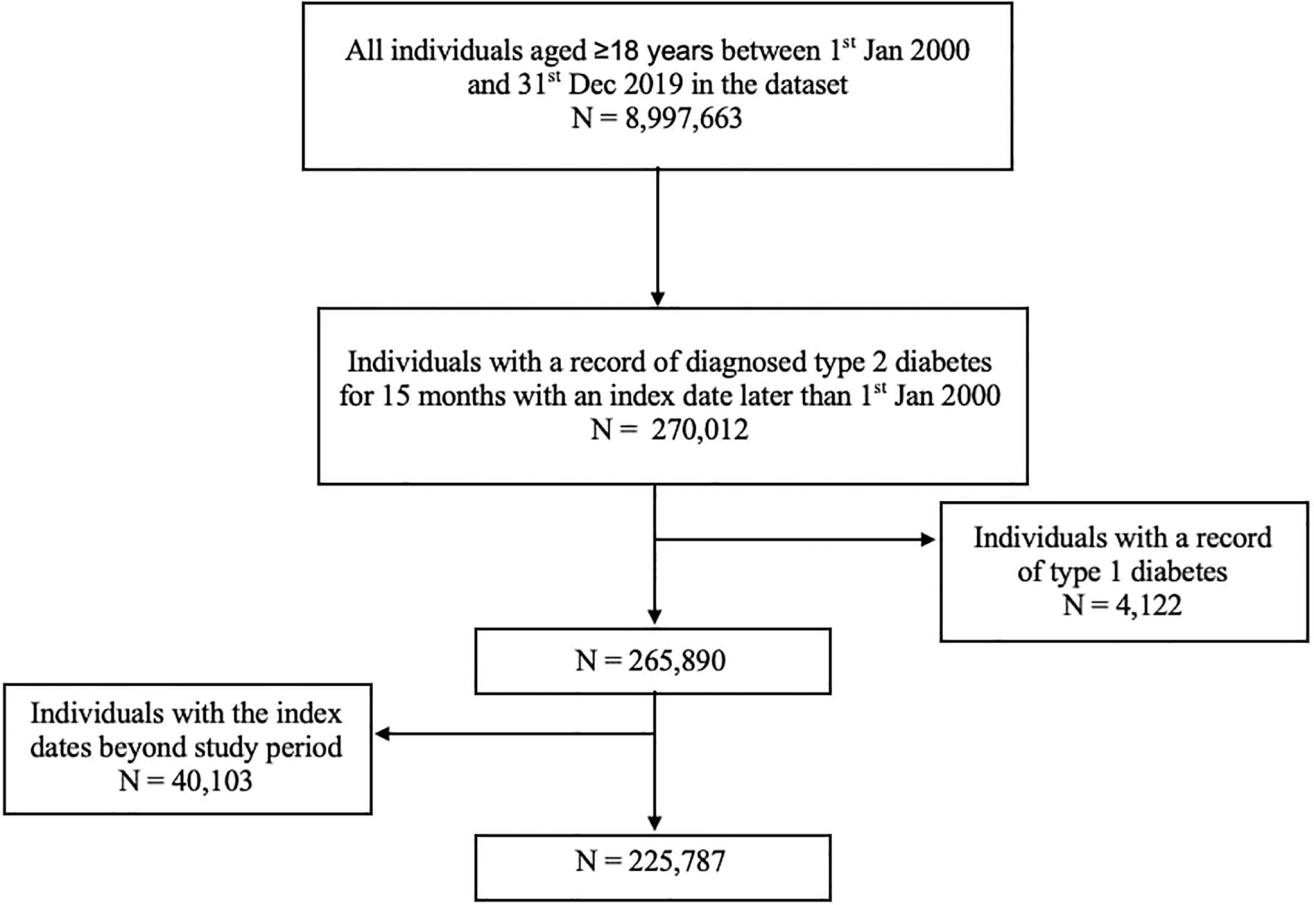
Study flowchart.

In total, 225,787 individuals who had been newly diagnosed with type 2 diabetes were included in the study with 77,346 (34%), 14,929 (7%), and 2,808 (1%) at low, moderate, and high risk of DFD, respectively. There were 1,118 (0.05%) individuals who declined the foot examination and 129,586 (57%) who had no recording of foot risk. **Figure 1** described the flow of the study population selection. Baseline characteristics are summarized in **Table 1**. The population was mostly male (55.7%) and over 50 years old (83.6%). Mean (SD) BMI and HbA1c were observed to be similar in three risk groups (BMI: 31.8 kg/m^2^ [6.6] vs. 31.8 kg/m^2^ [7.1] vs. 31.6 kg/m^2^ [7.4] and HbA1c: 51.5 mmol/mol [12.7] vs. 51.5 mmol/mol [12.5] vs. 52.5 mmol/mol [13.2], respectively); 16.3% of the individuals were active smokers. Individuals who refused a foot examination were more likely to be from more deprived groups. The prevalence of hypertension at baseline was higher in both the moderate-risk (59.3%) and high-risk (59.9%) groups compared with those in the low-risk group (51.6%).

**Table 1:** Baseline characteristics of new-onset type 2 diabetes individuals at risk of DFD.

	Low risk	Moderate risk	High risk	Foot examination declined	No recording
**Population, *n***	77,346	14,929	2,808	1,118	129,586
**Age, year, mean (SD)**	61.6 (12.8)	68.3 (12.6)	69.9 (12.6)	60.9 (14.3)	63.7 (13.1)
**Age, years, *n* (%)**					
18–29	623 (0.8)	36 (0.2)	9 (0.3)	12 (1.1)	756 (0.6)
30–39	3,162 (4.1)	208 (1.4)	28 (1.0)	64 (5.7)	4,452 (3.5)
40–49	10,865 (14.0)	1,051 (7.0)	165 (5.9)	197 (17.6)	15,334 (11.8)
50–59	19,695 (25.5)	2,516 (16.9)	441 (15.7)	275 (24.6)	28,791 (22.2)
60–69	22,130 (28.6)	3,909 (26.2)	682 (24.3)	259 (23.2)	36,204 (27.9)
≥70	20,871 (27.0)	7,209 (48.3)	1,483 (52.8)	311 (27.8)	44,049 (34.0)
**Sex, *n* (%)**					
Male	43,379 (56.1)	8,270 (55.4)	1,679 (59.8)	639 (57.2)	71,771 (55.4)
Female	33,967 (43.9)	6,659 (44.6)	1,129 (40.2)	479 (42.8)	57,815 (44.6)
**Ethnicity, *n* (%)**					
White	36,837 (47.6)	7,324 (49.1)	1,353 (48.2)	554 (49.5)	53,007 (40.9)
Black, African, Caribbean, or Black British	1,301 (1.7)	130 (0.9)	19 (0.7)	15 (1.3)	1,534 (1.2)
Asian or Asian British	3,051 (3.9)	244 (1.6)	24 (0.8)	23 (2.1)	3,700 (2.9)
Mixed or Multiple ethnic groups	737 (1.0)	52 (0.4)	2 (0.1)	5 (0.5)	657 (0.5)
Other ethnic group	246 (0.3)	22 (0.1)	3 (0.1)	1 (0.1)	245 (0.2)
Missing	35,175 (45.5)	7,157 (47.9)	1,407 (50.1)	520 (46.5)	70,443 (54.3)
**Townsend Score**					
1 (Least deprived)	13,197 (17.1)	2,409 (16.1)	410 (14.6)	142 (12.7)	25,552 (19.7)
2	13,246 (17.1)	2,426 (16.3)	498 (17.7)	140 (12.5)	23,655 (18.3)
3	13,801 (17.8)	2,764 (18.5)	514 (18.3)	188 (16.8)	24,177 (18.7)
4	12,931 (16.7)	2,669 (17.9)	534 (19.1)	195 (17.4)	22,442 (17.3)
5 (Most deprived)	9,480 (12.3)	2,241 (15.0)	469 (16.7)	228 (20.4)	17,110 (13.2)
Missing	14,691 (19.00)	2,420 (16.2)	383 (13.6)	225 (21.2)	16,650 (12.8)
**Smoking, *n* (%)**					
Non-smoker	37,903 (49.0)	6,347 (42.5)	1,116 (39.7)	485 (43.4)	60,476 (46.7)
Ex-smoker	27,416 (35.4)	5,928 (39.7)	1,164 (41.5)	365 (32.6)	46,679 (36.0)
Smoker	12,016 (15.5)	2,653 (17.7)	528 (18.8)	266 (23.8)	21,257 (16.4)
Missing	11 (0.01)	1 (0.01)	0 (0.0)	2 (0.2)	1,174 (0.9)
**BMI, kg/m^2^, mean (SD)**	31.8 (6.6)	31.8 (7.1)	31.6 (7.4)	33.1 (7.7)	31.0 (6.4)
**BMI, kg/m^2^, *n* (%)**					
Underweight <18.5	275 (0.4)	103 (0.7)	25 (0.9)	6 (0.6)	759 (0.6)
Normal weight 18.5 to <25	9,177 (11.9)	2,046 (13.7)	414 (14.7)	118 (10.5)	18,910 (14.6)
Overweight 25 to <30	24,663 (31.9)	4,533 (30.4)	866 (30.8)	294 (26.3)	43,608 (33.6)
Obesity class I 30 to <35	22,157 (28.6)	4,152 (27.8)	738 (26.3)	297 (26.6)	35,205 (27.2)
Obesity class II 35 to <40	11,911 (15.4)	2,169 (14.5)	358 (12.8)	191 (17.1)	16,911 (13.0)
Obesity class III ≥40	8,209 (10.6)	1,687 (11.3)	340 (12.1)	178 (15.9)	10,866 (8.4)
Missing	954 (1.2)	239 (1.6)	67 (2.4)	34 (3.0)	3,327 (2.6)
**HbA1c, mmol/mol, mean (SD)**	51.5 (12.7)	51.5 (12.5)	52.5 (13.2)	54.3 (15.2)	52.0 (13.8)
**HbA1c, mmol/mol, *n* (%)**					
≤47.5	31,117 (40.2)	5,754 (38.5)	983 (35.0)	266 (23.8)	12,045 (9.3)
47.5–58.5	28,833 (37.3)	5,672 (38.00)	1,026 (36.5)	242 (21.7)	11,672 (9.0)
58.5–69.4	7,218 (9.3)	1,323 (8.9)	289 (10.3)	99 (8.9)	3,176 (2.5)
>69.4	5,421 (7.0)	980 (6.6)	213 (7.6)	96 (8.6)	2,375 (1.8)
Missing or implausible	4,757 (6.2)	1,200 (8.0)	297 (10.6)	415 (37.1)	100,318 (77.4)
**CVD, *n* (%)**					
Hypertension	39,922 (51.6)	8,853 (59.3)	1,683 (59.9)	623 (55.7)	73,934 (57.1)
Atrial fibrillation	4,271 (5.5)	1,912 (12.8)	525 (18.7)	66 (5.9)	8,678 (6.7)
Heart failure	2,212 (2.9)	1,019 (6.8)	290 (10.3)	50 (4.5)	5,628 (4.3)
Ischemic heart disease	11,496 (14.9)	3,555 (23.8)	799 (28.5)	198 (17.7)	25,472 (19.7)
Stroke/TIA	4,708 (6.1)	1,794 (12.0)	452 (16.1)	102 (9.1)	10,337 (8.0)

DFD, diabetic foot disease; TIA, transient ischemia attack.

Mortality Among Individuals at Risk of DFD. Among the study population, 34,061 (15.1%) died during the study period. A total of 4,322 (5.6%), 1,904 (12.8%), and 549 (19.6%) deaths occurred in those who were at low, moderate, and high risk of DFD, respectively. Among individuals who declined foot examination and those with no recording, there were 178 (15.9%) and 27,108 (20.9%) deaths, respectively.

### Association Between Risk of DFD and Mortality in Individuals With Newly Diagnosed Type 2 Diabetes

**Figure 2:**
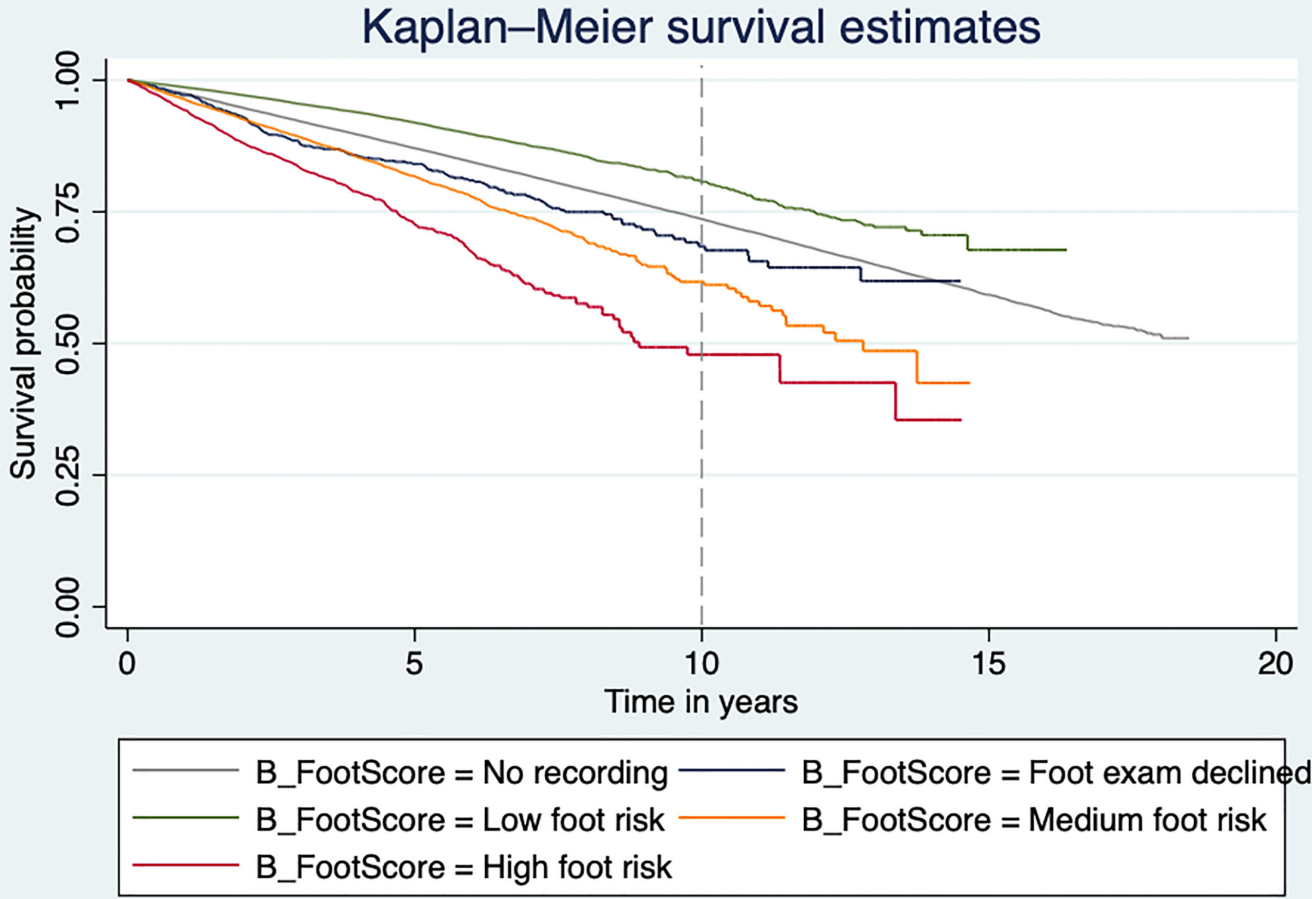
Kaplan–Meier plot showing the mortality risk of type 2 diabetes individuals at risk of DFD.

**Table 2** shows the unadjusted and adjusted HRs from the Cox regression model. Compared with low risk of DFD, the unadjusted hazards of mortality were higher for moderate and high DFD risk groups (HR 2.42, 95% CI [2.29, 2.55], *p* < 0.001; HR 3.77, 95% CI [3.45, 4.11], *p* < 0.001, respectively). **Figure 2** shows the Kaplan–Meier curve for the mortality rate related to foot risk. The graph lines start to separate from the beginning, representing the significant reduction in mortality in the patient group with low risk compared to moderate and high risk. The difference between the curves was statistically significant (*p* < 0.001).

**Table 2:** Unadjusted and adjusted HR of mortality rate in new-onset type 2 diabetes individuals at risk of DFD.

	Low DFD risk	Moderate DFD risk	High DFD risk	Foot examination declined	No recording
Population, *n*	77,346	14,929	2,808	1,118	129,586
Death, *n*	4,322	1,904	549	178	27,108
Person-years	253,883.4	46,511.5	8,586.0	4,827.8	887,710.2
Crude IRR	17.0	40.9	63.9	36.9	30.5
Unadjusted HR (95% CI), *p*-value	1	2.42 (2.29, 2.55), <0.001	3.77 (3.45, 4.11), <0.001	2.06 (1.78, 2.40), <0.001	1.62 (1.57, 1.67), <0.001
Adjusted HR (95% CI), *p*-value	1	1.50 (1.42, 1.58), <0.001	2.01 (1.84, 2.20), <0.001	1.75 (1.51, 2.04), <0.001	1.25 (1.20, 1.30), <0.001

IRR, Incidence Rate/1000 person-years.

Adjusted for age, sex, Townsend score, ethnicity, smoking, BMI, CVD event, HbA1c level, anti-diabetic medication use, lipid drug use, and hypertension.

After adjusting for age, sex, Townsend score, ethnicity, smoking status, baseline BMI, CVD, baseline HbA1c, and medications, those with a moderate risk of DFD had 1.5 times greater risk of mortality (HR 1.50, 95% CI [1.42, 1.58], *p* < 0.001), and those with a high risk of DFD had double the risk of mortality (HR, 2.01, 95% CI [1.84, 2.20], *p* < 0.001) compared to those with a low risk of DFD. In addition, those who declined a foot examination or who had no recording were also 75% and 25%, respectively, more likely to die than those with a low risk of DFD (HR 1.75, 95% CI [1.51, 2.04], *p* < 0.001; HR 1.25, 95% CI [1.20, 1.30], *p* < 0.001).

### Factors Associated With Increased Mortality

Age, Townsend score, smoking status, CVD events (AF, heart failure, IHD, and stroke), HbA1c, and antidiabetic drug use were associated with increased mortality (**Table 3**). Individuals who were recorded as being more deprived experienced higher mortality risk. Black, African, Caribbean, or Black British (HR 0.69, 95% CI [0.59, 0.80]); Asian or British Asian (HR 0.61, 95% CI [0.54, 0.68]); and mixed or multiple ethnic groups (HR 0.69, 95% CI [0.54, 0.90]) were less likely to die than those from the White ethnic group population. Former and current smokers had a higher risk of mortality compared to those who never smoked (HR 1.25, 95% CI [1.22, 1.28]; HR 1.77, 95% CI [1.72, 1.83], respectively). Moreover, hazards of mortality were lower in individuals with obesity and significantly higher in those who were underweight compared to those with normal weight (BMI 18.5 to <25 kg/m^2^). Individuals who were categorized as obesity class II (35 to <40 kg/m^2^) had approximately 25% reduced risk of mortality compared to those with a healthy weight with 0.75 (95% CI [0.72, 0.78]), while underweight individuals (<18.5 kg/m^2^) had twice the risk of dying (2.24, 95% CI [2.04, 2.45]). A close association was noted between increasing baseline Hba1c and risk of mortality. Those prescribed lipid-lowering drugs had a 29% lower risk of death (HR 0.71, 95% [0.69, 0.73]), compared to those not prescribed.

**Table 3:** Factors associated with mortality in new-onset type 2 diabetes individuals at risk of DFD .

	Death	HR (95% CI)
**Age, years**		
18–29	19/1,436	1.00
30–39	143/7,914	1.28 (0.79, 2.06)
40–49	828/27,612	2.26 (1.44, 3.57)
50–59	3,093/51,718	4.52 (2.88, 7.10)
60–69	7,830/63,184	9.11 (5.80, 14.30)
≥70	22,148/73,923	26.15 (16.66, 41.04)
**Sex**		
Male	18,777/125,738	1.00
Female	15,284/100,049	0.92 (0.89, 0.94)
**Townsend score**		
1 (Least deprived)	5,555/41,710	1.00
2	6,038/39,965	1.14 (1.10, 1.18)
3	6,323/41,444	1.21 (1.17, 1.26)
4	6,495/38,771	1.34 (1.29, 1.39)
5 (Most deprived)	4,968/29,528	1.42 (1.37, 1.48)
Missing	4,682/34,369	1.20 (1.15, 1.25)
**Ethnicity**		
White	12,601/99,075	1.00
Black, African, Caribbean, or Black British	157/2,999	0.69 (0.59, 0.80)
Asian or Asian British	297/7,041	0.61 (0.54, 0.68)
Mixed or Multiple ethnic groups	59/1,453	0.69 (0.54, 0.90)
Other ethnic group	28/517	0.75 (0.51, 1.08)
Missing	20,919/114,702	1.42 (1.39, 1.45)
**Smoking**		
Non-smoker	13,445/106,327	1.00
Ex-smoker	14,198/81,552	1.25 (1.22, 1.28)
Smoker	6,014/36,720	1.77 (1.72, 1.83)
Missing	404/1,188	0.99 (0.89, 1.10)
**BMI**		
Normal weight 18.5 to <25	7,258/30,665	1.00
Underweight <18.5	516/1,168	2.24 (2.04, 2.45)
Overweight 25 to <30	11,767/73,964	0.73 (0.71, 0.75)
Obesity class I 30 to <35	7,838/62,549	0.72 (0.70, 0.75)
Obesity class II 35 to <40	3,260/31,540	0.75 (0.72, 0.78)
Obesity class III ≥40	2,034/21,280	0.99 (0.95, 1.05)
Missing	1,388/4,621	1.56 (1.47, 1.65)
**CVD**		
Non-CVD	17,393/164,021	1.00
CVD	16,668/61,766	1.87 (1.83, 1.91)
**HbA1c**		
≤47.5	3,990/50,165	1.00
47.51–58.5	3,690/47,445	1.00 (0.96, 1.05)
58.51–69.4	929/12,105	1.13 (1.05, 1.21)
>69.41	665/9,085	1.37 (1.26, 1.49)
Missing or implausible	24,787/106,987	1.02 (0.98, 1.06)
**Antidiabetic Medication use**		
No medication or metformin	24,064/177,148	1.00
Other medication	8,410/41,666	1.37 (1.34, 1.41)
Insulin	1,587/6,973	2.09 (1.99, 2.20)
**Lipid drug use**		
Non-user	10,899/67,425	1.00
Lipid drug user	23,162/158,362	0.71 (0.69, 0.73)
**Hypertension**		
Non-hypertension event	12,522/100,772	1.00
Hypertension event	21,539/125,015	1.01 (0.99, 1.04)

DFD, diabetic foot disease; HR, hazard ratio.

### Sensitivity Analyses

In the sensitivity analysis where the index date was set to 30 months after the diagnosis of type 2 diabetes, 190,422 people (among whom 28,065 died) with a diagnosis of type 2 diabetes were included in the analysis. There was a similar trend between DFD risk and mortality with an HR of 1.46 (95% CI [1.39, 1.54]) and an HR of 2.04 (95% CI [1.89, 2.21]) in groups with a moderate and high risk of DFD, respectively, compared to the group with a low risk of DFD (**Supplementary Tables 1**, **2**).

After excluding those with incomplete data for BMI, Townsend score, and smoking status in the main dataset (index date of 15 months post diagnosis), 186,862 individuals were available for analysis (28,015 deaths). Individuals with moderate and high risk of DFD remained at higher risk of death than those at low risk of DFD (**Supplementary Table 3**, HR 1.47, 95% CI [1.39, 1.57]; HR 2.00, 95% CI [1.81, 2.20], respectively).

## Discussion

In this large cohort of adults with type 2 diabetes, we found that the risk of DFD is significantly associated with increased risk of death. Individuals who declined foot examination or who had no record also had increased mortality risk. The findings highlight the importance of assessing foot risk as it not only identifies patients at risk of DFU but also mortality. In addition, age, deprivation status, smoking status, poor glycemic control, and presence of CVD also contributed to an increased risk of mortality.

Elevated mortality in patients with a high risk of DFD defined as history of foot ulcer, Charcot arthropathy, or lower extremity amputation has previously been demonstrated (7, 8). The findings in this study show a similar trend of elevated mortality in those with a moderate/high foot risk among people with newly diagnosed type 2 diabetes. In addition, the present study included those who declined a foot examination, and those who had no recording of a foot examination; both groups had a greater risk of death compared to those in the low DFD risk group. There was an increased rate of refusal of foot examination in more deprived groups, which may further contribute to health inequalities in this group, suggesting that specific strategies to engage more socially deprived groups after a diagnosis of type 2 diabetes are needed (24). Foot protection service, including assessing the biomechanical status of the feet and the vascular status of the lower limbs, and providing specialist footwear and orthoses, in those with elevated DFD risk at the time of diagnosis of type 2 diabetes may help prevent progression of DFD such as foot ulcer and limb amputation, reducing morbidity and the direct and indirect health costs for diabetes management (6).

The lower risk of mortality in patients with obesity compared to those with a BMI in the normal BMI range has been demonstrated in prior studies (27, 28). It is possible that increased mobilization of endothelial progenitor cells leading to better vascular function protects severely obese patients (29). Another potential explanation is the nutritional status and the effective treatment for certain conditions such as hyperlipidemia, avoiding progression of foot disorder in later stage (29, 30). Smoking also increased the risk of mortality, which is consistent with smoking increasing the risk of PVD (31). People from ethnic minorities also had a lower risk of mortality compared to the White ethnic group, which has been shown in a previous study on DFD (24). Genetics, microcirculation preservation, lower smoking frequency, and less alcohol intake possibly cause the lower rate of diabetic foot problem among ethnic minorities compared to the White ethnic group, eventually reducing the mortality (32, 33).

Mechanisms that increase the risk of mortality in patients with type 2 diabetes and moderate or high foot risk are multifactorial. PAD is a marker for systemic vascular disease and associated with an excess risk of CVD events and death (34). Ischemia caused by PAD predicts the risk of low-extremity amputation, particularly in people with diabetes, leading to a greater risk of mortality (34, 35). Autonomic neuropathy is associated with the development of DFU, and is a possible risk factor for mortality in patients with diabetes (36, 37, 38). Moreover, it has been shown that peripheral neuropathy was independently associated with incident CVD events and linked to an increased risk of mortality (16). Taken together, this may explain the high risk of mortality among type 2 diabetes patients who were at risk of DFD.

### Strengths and Limitations

This study has a number of strengths ensuring a high-quality study with reliable results including a large sample size. Patients in IMRD are broadly representative of the UK population, and thus, these results should be generalizable. Sensitivity analysis where follow-up started 30 months after the diagnosis of type 2 diabetes allows sufficient time for the exposure to be recorded, and this also showed an increased risk of death for higher DFD risk. Further research is needed to explore the underlying reasons for declining foot examination/absence of recorded data, and the elevated mortality identified in these groups.

Limitations include missing data for some covariates; however, it was a small proportion of the total dataset, and the sensitivity analysis excluding those with missing data showed a consistent association. Renal replacement therapy was not considered as high risk of DFD although it was suggested to be at high risk in NICE guidelines (6). However, there were only a small number of participants (≤0.2%) recorded with this therapy, which did not influence the reliability of the results. We did not have information on the cause of death, and this should be considered in further studies to determine important risk factors that should be targeted to reduce the risk of death among people with newly diagnosed type 2 diabetes who are at risk of DFD.

## Conclusion

In conclusion, individuals with new-onset type 2 diabetes who are at increased risk of DFD experienced a higher risk of death compared to those at low risk of DFD. This key finding of the association of DFD risk and mortality highlights the importance of foot risk assessment in people with type 2 diabetes, and a potential role for early identification and management of at-risk patients. The increased proportion of individuals declining foot examination in more deprived groups and the associated increased mortality are a particular concern, as they may further exacerbate health inequalities; the development of strategies that target these groups is warranted.

## Data Availability Statement

The datasets presented in this article are not readily available because the study is based on The Health Improvement Network (THIN) database licenced by IQVIA, in which individual patient data are not allowed to be shared to the public. Researchers may apply for individual patient data access at https://www.iqvia.com/contact. Requests to access the datasets should be directed to Researchers may apply for individual patient data access at https://www.iqvia.com/contact.

## Author Contributions

ZW, KN, FC, and JH contributed to study design, acquisition of data, and statistical analysis. ZW wrote the manuscript and FC, JH, KN, AT, WH, NT, JW, and CS critically revised the manuscript. ZW is the guarantor of this work. All authors contributed to this work. All authors approved the final version of submission.

## Conflict of Interest

The authors declare that the research was conducted in the absence of any commercial or financial relationships that could be construed as a potential conflict of interest.

## Publisher’s Note

All claims expressed in this article are solely those of the authors and do not necessarily represent those of their affiliated organizations, or those of the publisher, the editors and the reviewers. Any product that may be evaluated in this article, or claim that may be made by its manufacturer, is not guaranteed or endorsed by the publisher.

## Supplementary Material

Supplementary Table 1 Baseline characteristics of new-onset type 2 diabetes individuals at risk of DFD (30 months)

Supplementary Table 2 HR of mortality rate in new-onset type 2 diabetes individuals at risk of DFD and factors associated with mortality (30 months)

Supplementary Table 3 Sensitivity analysis excluding missing data for Townsend score, smoking, and BMI

## Abbreviations

AF, Atrial fibrillation; CPRD, Clinical Practice Research Datalink; DExtER, Data extraction for epidemiological research; DFD, Diabetic foot disease; DPN, Diabetic peripheral neuropathy; DPP-4, Dipeptidyl peptidase-4; GLP-1, Glucagon-like peptide 1; HRQOL, Health-related quality of life; IMRD, IQVIA Medical Research Data; IHD, Ischemic heart disease; IRR, Incidence rate; MREC, Multi-centre Research Ethics Committee; NICE, National Institute for Health and Care Excellence; PVD, Peripheral vascular disease; QOF, Quality Outcomes Framework; SF-36, Medical Outcomes Study Short Form 36 Healthy Survey; SGLT-2, Sodium-glucose co-transporter-2; THIN, The Health Improvement Network; TIA, Transient ischemic attacks.
