# Supplementary material for: Diabetic Foot Risk Classification at the Time of Type 2 Diabetes Diagnosis and Subsequent Risk of Mortality: A Population-Based Cohort Study
Source: Front Endocrinol (Lausanne). 2022 Jul 11;13:888924. doi: 10.3389/fendo.2022.888924 (PMC9309507; doi:10.3389/fendo.2022.888924)
Supplement: Supplementary Table 1 — Baseline characteristics of new-onset type 2 diabetes individuals at risk of DFD (30 months) [file Table_1.docx]

**Supplementary Tables**

**STable 1. Baseline characteristics of new-onset type 2 diabetes individuals at risk of DFD (30 months)**

|  | **Low Risk** | **Moderate Risk** | **High Risk** | **Foot Examination Declined** | **No Recording** |
| --- | --- | --- | --- | --- | --- |
| **Population, n** | 70,708 | 18,068 | 3,678 | 1,270 | 96,698 |
| **Sex, n (%)** |  |  |  |  |  |
| Male | 39,893 (56.42) | 9,910 (54.85) | 2,184 (59.38) | 707 (55.67) | 53,377 (55.20) |
| Female | 30,815 (43.58) | 8,158 (45.15) | 1,494 (40.62) | 563 (44.33) | 43,321 (44.80) |
|  |  |  |  |  |  |
| **Age, year, mean (SD)** | 62.3 (12.5) | 68.8 (12.3) | 70.6 (12.4) | 62.4 (13.9) | 64.8 (12.9) |
|  |  |  |  |  |  |
| **Age, year, n (%)** |  |  |  |  |  |
| 18-29 | 410 (0.6) | 33 (0.2) | 8 (0.2) | 9 (0.7) | 380 (0.4) |
| 30-39 | 2,432 (3.4) | 209 (1.2) | 31 (0.8) | 50 (3.9) | 2,596 (2.7) |
| 40-49 | 9,076 (12.8) | 1,115 (6.2) | 190 (5.2) | 194 (15.3) | 10,206 (10.5) |
| 50-59 | 17,956 (25.4) | 2,989 (16.5) | 513 (14.0) | 325 (25.6) | 20,378 (21.1) |
| 60-69 | 20,888 (29.6) | 4,774 (26.4) | 915 (24.8) | 313 (24.7) | 27,305 (28.2) |
| ≥70 | 19,946 (28.2) | 8,948 (49.5) | 2,021 (55.0) | 379 (29.8) | 35,833 (37.1) |
|  |  |  |  |  |  |
| **Ethnicity, n (%)** |  |  |  |  |  |
| White | 33,023 (46.70) | 8,696 (48.13) | 1,760 (47.85) | 597 (47.01) | 39,963 (41.33) |
| Black, African,  Caribbean or Black British | 1,147 (1.62) | 163 (0.90) | 23 (0.63) | 12 (0.94) | 1,073 (1.11) |
| Asian or Asian British | 2,717 (3.84) | 330 (1.83) | 39 (1.06) | 23 (1.01) | 2,665 (2.76) |
| Mixed or Multiple ethnic  groups | 590 (0.83) | 61 (0.34) | 6 (0.16) | 7 (0.55) | 474 (0.49) |
| Other ethnic group | 246 (0.32) | 22 (0.15) | 3 (0.11) | 1 (0.09) | 245 (0.19) |
| Missing | 33,031 (46.71) | 8,796 (48.68) | 1,846 (50.19) | 631 (49.69) | 52,334 (54.12) |
|  |  |  |  |  |  |
| **Townsend Score** |  |  |  |  |  |
| 1 (Least deprived) | 12,286 (17.38) | 2,955 (16.35) | 526 (14.30) | 168 (13.23) | 19,479 (20.14) |
| 2 | 12,295 (17.39) | 2,995 (16.58) | 641 (17.43) | 191 (15.04) | 17,807 (18.42) |
| 3 | 12,672 (17.92) | 3,322 (18.39) | 690 (18.76) | 196 (15.43) | 18,129 (18.75) |
| 4 | 11,810 (16.70) | 3,230 (17.88) | 693 (18.84) | 235 (18.50) | 16,703 (17.27) |
| 5 (Most deprived) | 8,515 (12.04) | 2,631 (14.56) | 627 (17.05) | 257 (20.24) | 12,750 (13.19) |
| Missing | 13,130 (18.57) | 2,935 (16.24) | 501 (13.62) | 223 (17.56) | 11,830 (12.23) |
|  |  |  |  |  |  |
| **Smoking, n (%)** |  |  |  |  |  |
| Non-smoker | 34,875 (49.32) | 7,772 (43.02) | 1,517 (41.25) | 553 (43.54) | 45,589 (47.15) |
| Ex-smoker | 25,173 (35.60) | 7,246 (40.10) | 1,489 (40.48) | 412 (32.44) | 35,551 (36.76) |
| Smoker | 10,655 (15.07) | 3,049 (16.88) | 671 (18.24) | 301 (23.70) | 15,141 (15.66) |
| Missing | 5 (0.01) | 1 (0.01) | 1 (0.00) | 4 (0.31) | 417 (0.43) |
|  |  |  |  |  |  |
| **BMI, kg/m^2^, mean (SD)** | 31.8 (6.5) | 31.7 (7.0) | 31.5 (7.1) | 32.6 (7.4) | 31.00 (6.4) |
|  |  |  |  |  |  |
| **BMI, kg/m^2^, n (%)** |  |  |  |  |  |
| Underweight <18.5 | 250 (0.35) | 110 (0.61) | 34 (0.92) | 4 (0.31) | 581 (0.6) |
| Normal weight 18.5 to <25 | 8,331 (11.8) | 2,481 (13.7) | 556 (15.1) | 152 (12.0) | 14,150 (14.6) |
| Overweight 25 to <30 | 22,671 (32.1) | 5,603 (31.0) | 1,107 (30.1) | 353 (27.8) | 32,857 (34.0) |
| Obesity class I 30 to <35 | 20,742 (29.3) | 5,030 (27.8) | 1,039 (28.3) | 351 (27.6) | 26,648 (27.6) |
| Obesity class II 35 to <40 | 10,972 (15.5) | 2,652 (14.7) | 485 (13.2) | 206 (16.2) | 12,806 (13.24) |
| Obesity class III ≥40 | 7,200 (10.2) | 2,007 (11.1) | 399 (10.9) | 176 (13.9) | 8,056 (8.3) |
| Missing | 542 (0.8) | 185 (1.0) | 58 (1.6) | 28 (2.2) | 1,600 (1.7) |
|  |  |  |  |  |  |
| **HbA1c, mmol/mol, mean (SD)** | 52.9 (13.5) | 52.3 (12.9) | 53.0 (14.2) | 56.3 (18.3) | 52.7 (13.9) |
|  |  |  |  |  |  |
| **HbA1c, mmol/mol, n (%)** |  |  |  |  |  |
| ≤ 47.5 | 25,380 (35.89) | 6,547 (36.24) | 1,283 (34.88) | 245 (19.29) | 8,047 (8.32) |
| 47.5-58.5 | 26,372 (37.30) | 6,818 (37.74) | 1,298 (35.29) | 224 (17.64) | 8,929 (9.23) |
| 58.5-69.4 | 8,104 (11.46) | 1,830 (10.13) | 381 (10.36) | 104 (8.19) | 2,573 (2.66) |
| > 69.4 | 6,227 (8.81) | 1,422 (7.87) | 306 (8.32) | 111 (8.74) | 1,928 (1.99) |
| Missing or implausible | 4,625 (6.54) | 1,451 (8.03) | 410 (11.15) | 586 (46.14) | 75,221 (77.79) |
|  |  |  |  |  |  |
| **CVD, n (%)** |  |  |  |  |  |
| Hypertension | 38,460 (54.39) | 11,012 (60.95) | 2,295 (62.40) | 756 (59.53) | 58,667 (60.67) |
| Atrial fibrillation | 3,825 (5.41) | 2,208 (12.22) | 668 (18.16) | 91 (7.17) | 6,794 (7.03) |
| Heart failure | 1,979 (2.80) | 1,188 (6.58) | 376 (10.22) | 65 (5.12) | 4,363 (4.51) |
| Ischemic heart disease | 10,743 (15.19) | 4,308 (23.84) | 1,062 (28.87) | 261 (20.55) | 20,432 (21.13) |
| Stroke/TIA | 4,383 (6.20) | 2,167 (11.99) | 598 (16.26) | 131 (10.31) | 8,129 (8.41) |
| DFD=diabetic foot disease; TIA = transient ischemia attack. | | | | | |

**STable 2. HR of mortality rate in new-onset type 2 diabetes individuals at risk of DFD** **and factors associated with mortality (30 Months)**

|  | **HR (95% CI)** |
| --- | --- |
| **Unadjusted** |  |
| **DFD risk** |  |
| Low DFD risk | 1.00 |
| Moderate DFD risk | 2.32 (2.20, 2.44) |
| High DFD risk | 3.77 (3.49, 4.08) |
| Foot examination declined | 2.20 (1.94, 2.50) |
| No recording | 1.65 (1.60, 1.71) |
| **Adjusted** |  |
| **DFD risk** |  |
| Low DFD risk | 1.00 |
| Moderate DFD risk | 1.46 (1.39, 1.54) |
| High DFD risk | 2.04 (1.89, 2.21) |
| Foot examination declined | 1.92 (1.69, 2.18) |
| No recording | 1.27 (1.22, 1.33) |
| **Age, year** |  |
| 18-29 | 1.00 |
| 30-39 | 1.15 (0.61, 2.15) |
| 40-49 | 2.32 (1.28, 4.21) |
| 50-59 | 4.38 (2.42, 7.92) |
| 60-69 | 8.82 (4.88, 15.95) |
| ≥70 | 25.39 (14.04, 45.89) |
|  |  |
| **Sex** |  |
| Male | 1.00 |
| Female | 0.92 (0.90, 0.94) |
|  |  |
| **Townsend score** |  |
| 1 (Least deprived) | 1.00 |
| 2 | 1.14 (1.09, 1.19) |
| 3 | 1.22 (1.18, 1.27) |
| 4 | 1.36 (1.31, 1.42) |
| 5 (Most deprived) | 1.43 (1.37, 1.50) |
| Missing | 1.20 (1.15, 1.26) |
|  |  |
| **Ethnicity** |  |
| White | 1.00 |
| Black, African, Caribbean or Black British | 0.73 (0.50, 0.89) |
| Asian or Asian British | 0.62 (0.55, 0.70) |
| Mixed or Multiple ethnic groups | 0.67 (0.50, 0.89) |
| Other ethnic group | 0.74 (0.49, 1.12) |
| Missing | 1.37 (1.34, 1.41) |
|  |  |
| **Smoking** |  |
| Non-smoker | 1.00 |
| Ex-smoker | 1.26 (1.22, 1.29) |
| Smoker | 1.74 (1.68, 1.80) |
| Missing | 1.03 (0.87, 1.23) |
|  |  |
| **BMI** |  |
| Normal weight 18.5-24.9 | 1.00 |
| Underweight <18.5 | 2.30 (2.09, 2.54) |
| Overweight 25-29.9 | 0.70 (0.67, 0.72) |
| Obesity class I 30-34.9 | 0.68 (0.66, 0.70) |
| Obesity class II 35-39.9 | 0.71 (0.68, 0.74) |
| Obesity class III ≥40 | 0.94 (0.89, 0.99) |
| Missing | 1.64 (1.51, 1.77) |
|  |  |
| **CVD** |  |
| Non-CVD | 1.00 |
| CVD | 1.86 (1.82, 1.91) |
|  |  |
| **HbA1c** |  |
| ≤ 47.5 | 1.00 |
| 47.51-58.5 | 0.90 (0.86, 0.94) |
| 58.51-69.4 | 0.94 (0.88, 1.01) |
| > 69.4 | 1.09 (0.99, 1.18) |
| Missing or implausible | 0.95 (0.91, 0.99) |
|  |  |
| **Antidiabetic Medication use** |  |
| No medication or metformin | 1.00 |
| Other medication | 1.28 (1.25, 1.32) |
| Insulin | 1.86 (1.77, 1.96) |
|  |  |
| **Lipid drug use** |  |
| Non-user | 1.00 |
| Lipid drug user | 0.67 (0.65, 0.69) |
|  |  |
| **Hypertension** |  |
| Non-hypertension event | 1.00 |
| Hypertension event | 1.03 (0.99, 1.05) |
| DFD=diabetic foot disease. | |

**STable 3. Sensitivity analysis excluding missing data for Townsend score, smoking, and BMI**

|  | **HR (95% CI)** |
| --- | --- |
| **Adjusted** |  |
| **DFD risk** |  |
| Low DFD risk | 1.00 |
| Moderate DFD risk | 1.47 (1.39, 1.57) |
| High DFD risk | 2.00 (1.81, 1.20) |
| Foot examination declined | 1.81 (1.52, 2.16) |
| No recording | 1.23 (1.17, 1.28) |
| **Age, year** |  |
| 18-29 | 1.00 |
| 30-39 | 1.30 (0.76, 2.23) |
| 40-49 | 2.26 (1.35, 3.76) |
| 50-59 | 4.46 (2.68, 7.42) |
| 60-69 | 8.90 (5.35, 14.77) |
| ≥70 | 25.31 (15.24, 42.04) |
|  |  |
| **Sex** |  |
| Male | 1.00 |
| Female | 0.91 (0.89, 0.93) |
|  |  |
| **Townsend score** |  |
| 1 (Least deprived) | 1.00 |
| 2 | 1.14 (1.10, 1.18) |
| 3 | 1.21 (1.17, 1.26) |
| 4 | 1.34 (1.29, 1.39) |
| 5 (Most deprived) | 1.43 (1.38, 1.49) |
|  |  |
| **Ethnicity** |  |
| White | 1.00 |
| Black, African, Caribbean or Black British | 0.68 (0.57, 0.81) |
| Asian or Asian British | 0.62 (0.54, 0.71) |
| Mixed or Multiple ethnic groups | 0.55 (0.39, 0.77) |
| Other ethnic group | 0.76 (0.51, 1.14) |
| Missing | 1.44 (1.40, 1.47) |
|  |  |
| **Smoking** |  |
| Non-smoker | 1.00 |
| Ex-smoker | 1.26 (1.23, 1.30) |
| Smoker | 1.77 (1.71, 1.84) |
|  |  |
| **BMI** |  |
| Normal weight 18.5-24.9 | 1.00 |
| underweight <18.5 | 2.20 (2.00, 2.42) |
| Overweight 25-29.9 | 0.73 (0.71, 0.75) |
| Obesity class I 30-34.9 | 0.71 (0.69, 0.74) |
| Obesity class II 35-39.9 | 0.74 (0.71, 0.77) |
| Obesity class III ≥40 | 0.97 (0.92, 1.03) |
|  |  |
| **CVD** |  |
| Non-CVD | 1.00 |
| CVD | 1.88 (1.83, 1.92) |
|  |  |
| **HbA1c** |  |
| ≤ 47.5 | 1.00 |
| 47.51-58.5 | 1.01 (0.96, 1.06) |
| 58.51-69.4 | 1.13 (1.04, 1.22) |
| > 69.4 | 1.35 (1.22, 1.48) |
| Missing or implausible | 1.02 (0.97, 1.06) |
|  |  |
| **Antidiabetic Medication use** |  |
| No medication or metformin | 1.00 |
| Other medication | 1.37 (1.33, 1.41) |
| Insulin | 2.12 (2.00, 2.24) |
|  |  |
| **Lipid drug use** |  |
| Non-user | 1.00 |
| Lipid drug user | 0.71 (0.69, 0.73) |
|  |  |
| **Hypertension** |  |
| Non-hypertension event | 1.00 |
| Hypertension event | 1.02 (0.99, 1.05) |
| DFD=diabetic foot disease. |  |
